# P05.68. Teaching an interprofessional approach to the management of musculoskeletal problems in primary care – a pilot study

**DOI:** 10.1186/1472-6882-12-S1-P428

**Published:** 2012-06-12

**Authors:** D Kopansky-Giles, J Peranson, S Reeves

**Affiliations:** 1 Canadian Memorial Chiropractic College and St. Michael's Hospital, Toronto, Ontario, Canada; 2 St. Michael's Hospital, Toronto, Canada

## Purpose

### This pilot had three aims

1) To determine if a 4-day modular program will enable a mixed-professional group of learners to develop and/or improve their competencies in collaborative MSK health care; 2) To determine if this increased the student’s confidence in being a collaborative health practitioner; 3) To determine effective facilitation strategies for enabling the acquisition of collaborative competencies by a mixed group of health science students.

## Methods

This was a mixed methods design. Pre-and-post program semi-structured focus groups with students were conducted to explore satisfaction with the program, and perceptions of program impact on the acquisition of collaborative competencies in MSK care. Key informant interviews with teachers were conducted to determine program content and the different educational approaches to be used. Inductive thematic analysis and triangulation of data sets was utilized to evaluate qualitative data. A new quantitative tool, the Health Professional Collaborative Competency Scale ^©^2011 was also administered to students. A one-sided paired t test was used to compare pre- versus post-test scores from the instrument under the hypothesis that the educational module improved post-test scores. The standard for statistical significance was p<0.05.

## Results

Thirteen students from 6 different programs and 5 institutions participated, their year of study within their program varying to some degree. Learners also had variable levels of MSK training/experience with IPE. The students were satisfied with the program and discussing their own experiences and ideas as well as to learn from other students. Twenty-two facilitators participated from 11 professions and felt that best teaching was thought to occur through role modeling.

## Conclusion

A 4-day interprofessional educational module can enhance the collaborative abilities of mixed health professional learners. Role modeling (by mentor facilitators) and discussion/interactive activities appear to be important key features of such programs.

